# Supplementary material for: Intraspecific variation in thermal tolerance differs between tropical and temperate fishes
Source: Sci Rep. 2021 Oct 28;11:21272. doi: 10.1038/s41598-021-00695-8 (PMC8553816; doi:10.1038/s41598-021-00695-8)
Supplement: Supplementary file 1 — Supplementary Information. [file 41598_2021_695_MOESM1_ESM.pdf]

Intraspecific variation in thermal tolerance differs between tropical and temperate fishes

Authors: J.J.H. Nati[1,2*], M.B.S. Svendsen[3], S. Marras[4], S.S. Killen[1], J.F. Steffensen[3], D.J. McKenzie[2], P. Domenici[4]

[1]Institute of Biodiversity, Animal Health and Comparative Medicine, University of Glasgow, Graham Kerr Building, Glasgow G12 8QQ, UK.

[2]MARBEC, Université de Montpellier, CNRS, IFREMER, IRD, 34000 Montpellier, France.

[3]Marine Biological Section, Department of Biology, University of Copenhagen, Strandpromenaden 5, 3000 Helsingør, Denmark.

[4]CNR-IAS, Italian National Research Council, Institute of Anthropic impacts and Sustainability in the Marine Environment, Torregrande, 09170, Oristano, Italy

Corresponding author:julienati3@gmail.com

Supplementary Methods and Results

1) **Model selection approach:** to test differences in intraspecific variation in CT_{max} between two relevant latitudinal groups, habitat types, hemisphere effect and methodology effects.

PGLS models from the most complex to the simple one:

Model 1 : $\log_{10}$ S.D. CT_{max} ~ α_0 + α_1 Latitudinal position + α_2 Habitat + α_3 delta Temperature + α_4 Hemisphere + α_5 Number of individuals used + α_6 mean CT_{max}+ α_7 Latitudinal position x delta Temperature + α_8 Latitudinal position x Hemisphere + α_9 Latitudinal position x Habitat + ε

Model 2 : $\log_{10}$ S.D. CT_{max} ~ α_0 + α_1 Latitudinal position + α_2 Habitat + α_3 delta Temperature + α_4 Hemisphere + α_5 Number of individuals used + α_6 mean CT_{max}+ α_7 Latitudinal position x delta Temperature + α_8 Latitudinal position x Habitat + ε

Model 3 : $\log_{10}$ S.D. CT_{max} ~ α_0 + α_1 Latitudinal position + α_2 Habitat + α_3 delta Temperature + α_4 Hemisphere + α_5 Number of individuals used + α_6 mean CT_{max}+ α_7 Latitudinal position x delta Temperature + ε

Model 4: $\log_{10}$ S.D. CT_{max} ~ α_0 + α_1 Latitudinal position + α_2 Habitat + α_3 delta Temperature + α_4 Hemisphere + α_5 Number of individuals used + α_6 mean CT_{max} + ε

Model 5 : $\log_{10}$ S.D. CT_{max} ~ α_0 + α_1 Latitudinal position + α_2 Habitat + α_3 delta Temperature + α_4 Hemisphere + α_5 Number of individuals used + ε

Model 6: $\log_{10}$ S.D. CT_{max} ~ α_0 + α_1 Latitudinal position + α_2 delta Temperature + α_3 Hemisphere + α_4 Number of individuals used + ε

Model 7: $\log_{10}$ S.D. CT_{max} ~ α_0 + α_1 Latitudinal position + α_2 delta Temperature + α_3 Hemisphere + ε

Model 8 : $\log_{10}$ S.D. CT_{max} ~ α_0 + α_1 delta Temperature + α_2 Hemisphere + ε

Model 9 : $\log_{10}$ S.D. CT_{max} ~ α_0 + α_1 Hemisphere + ε

Model 10 : $\log_{10}$ S.D. CT_{max} ~ α_0 + ε

Supplementary Table 1: PGLS model selection approach by AIC function on 203 fish species. Selected model is model 1 due to lowest AIC value.

N species	Models	df	AIC	ΔAIC
203	Model 1	10	266.1	
	Model 2	9	266.5	-0.5
	Model 3	8	267.6	-1.5
	Model 4	7	278.8	-12.7
	Model 5	6	277.3	-11.2
	Model 6	5	276.7	-10.6
	Model 7	4	279.5	-13.4
	Model 8	3	286.9	-20.8
	Model 9	2	315.4	-49.3
	Model 10	1	344.5	-78.4

2) Phylogenetic informed analysis on intraspecific variation of CT_{max} in 203 species

Model 1 : $\log_{10}$ S.D. CT_{max} ~ α_0 + α_1 Latitudinal position + α_2 Habitat + α_3 delta Temperature + α_4 Hemisphere + α_5 Number of individuals used + α_6 mean CT_{max}+ α_7 Latitudinal position x delta Temperature + α_8 Latitudinal position x Hemisphere + α_9 Latitudinal position x Habitat + ε

Supplementary Table 2: PGLS model summary, $F_{9,192} = 4.452$, $\lambda = 0.430$, $R^2 = 17.27$, $p < 0.001$ on 203 fish species.

N species	coefficients	estimates	s.e.	t values	p values
203	Intercept	0.212	0.312	0.681	0.497
	Tropical species	-0.72	0.252	-2.844	0.005
	Marine species	-0.198	0.073	-2.700	0.008
	Δ Temperature	0.01	0.007	1.523	0.13
	Southern hemisphere	0.028	0.089	0.32	0.75
	Individuals	0.001	0.002	0.553	0.581
	CT_{max}	-0.015	0.007	-2.17	0.031
	Tropical sp*ΔTemperature	0.05	0.020	2.266	0.025
	Tropical*Southern hemispehre	0.201	0.127	1.58	0.116
	Tropical*Marine	0.243	0.115	2.116	0.036

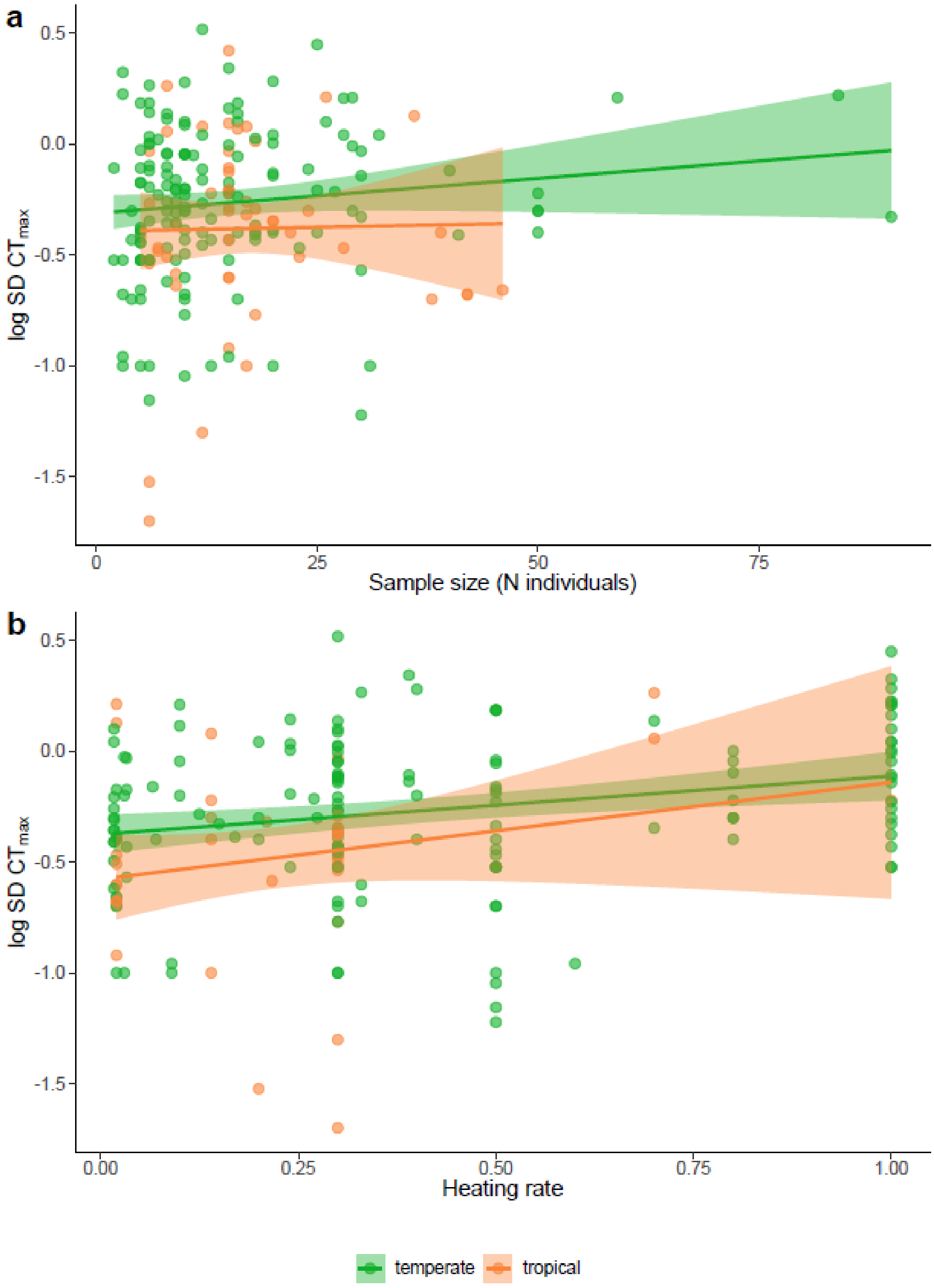

Figure S1| The effect of (a) the number of individuals and (b) heating rate (°C/min) used in each study on log_{10} transformed standard deviation of CT_{max} (log_{10} S.D. CT_{max}) divided by two latitudinal groups (tropical and temperate species). The shaded areas in the regression lines correspond to 95% of confidence interval.

3) GLM analysis on intraspecific variation CT_{max} in 203 species

Supplementary Table 3: GLM model summary on log_{10} S.D.CT_{max}, $F_{9,193} = 5.347$, $R^2 = 19.96$ $p < 0.001$ in 203 fish species.

N species	coefficients	estimates	s.e.	t values	p values
203	Intercept	0.158	0.255	0.617	0.538
	Tropical species	-0.728	0.26	-2.840	0.005
	Marine species	-0.194	0.061	-3.153	0.0019
	Δ Temperature	0.011	0.007	1.581	0.115
	Southern Hemisphere	-0.028	0.089	-0.314	0.754
	Individuals	0.001	0.002	0.680	0.497
	CT_{max}	-0.016	0.006	-2.626	0.009
	Tropical sp*ΔTemperature	0.04	0.02	2.149	0.033
	Tropical*Southern hemisphere	0.274	0.131	2.100	0.037
	Tropical*Marine	0.291	0.116	2.513	0.013

4) Phylogenetic informed analysis on intraspecific variation of CT_{max} in 186 species

Model 1 : $\log_{10}$ S.D. $CT_{max} \sim \alpha_0 + \alpha_1$ Latitudinal position + α_2 Habitat + $\alpha_3$delta Temperature+ α_4 Hemisphere + α_5 Number of individuals used + $\alpha_6$mean CT_{max}+ α_7 Heating rate (°C/min)+ α_8 Latitudinal position x delta Temperature + ε

Supplementary Table 4: PGLS model summary, $F_{10,174} = 4.242$, $\lambda = 0.427$, $R^2 = 19.6$, $p < 0.001$ on 186
fish species.

N species	coefficients	Estimates	s.e.	t values	p values
186	Intercept	0.247	0.32	0.773	0.441
	Tropical species	-0.75	0.263	-2.855	0.005
	Marine species	-0.175	0.074	-2.351	0.02
	Δ Temperature	0.005	0.008	0.631	0.529
	Southern Hemisphere	0.02	0.09	0.181	0.857
	Individuals	0.001	0.002	0.564	0.574
	CT_{max}	-0.016	0.007	-2.168	0.032
	Heating rate	0.203	0.083	2.433	0.016
	Tropical sp*ΔTemperature	0.055	0.021	2.678	0.008
	Tropical*Southern hemisphere	-0.018	0.187	-0.094	0.926

	Tropical*Marine	0.127	0.14	0.911	0.363

5) Supplementary Figures

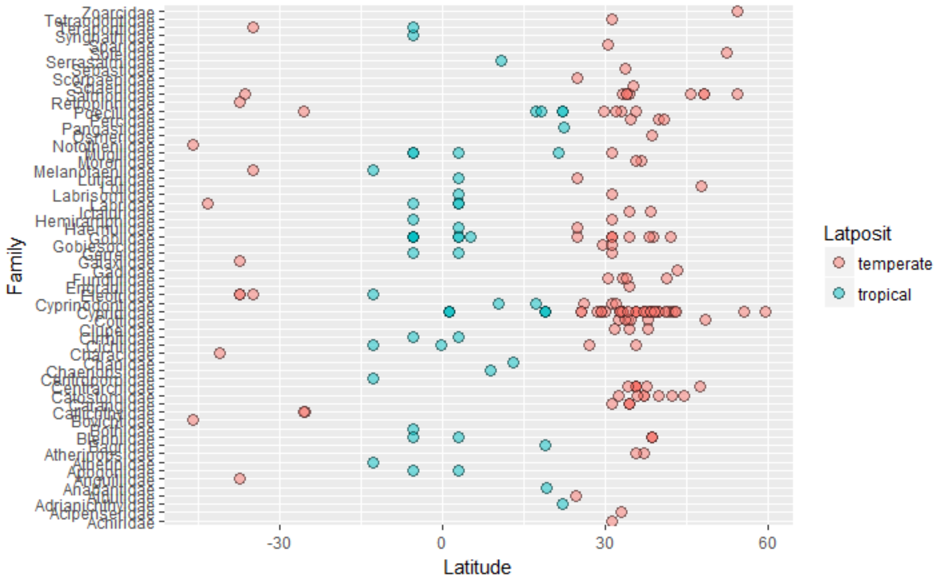

Figure S2| Representation of family across latitude divided in two latitudinal groups. red dot temperate species, blue dots in tropical species.
